# Causes and consequences of genomic instability in laminopathies: Replication stress and interferon response

**DOI:** 10.1080/19491034.2018.1454168

**Published:** 2018-05-07

**Authors:** Simona Graziano, Ray Kreienkamp, Nuria Coll-Bonfill,, Susana Gonzalo

**Affiliations:** Edward A. Doisy Department of Biochemistry and Molecular Biology, Saint Louis University School of Medicine, St. Louis, MO, USA

**Keywords:** Lamins, progeria, genomic instability, replication stress, interferon response

## Abstract

Mammalian nuclei are equipped with a framework of intermediate filaments that function as a karyoskeleton. This nuclear scaffold, formed primarily by lamins (A-type and B-type), maintains the spatial and functional organization of the genome and of sub-nuclear compartments. Over the past decade, a body of evidence has highlighted the significance of these structural nuclear proteins in the maintenance of nuclear architecture and mechanical stability, as well as genome function and integrity. The importance of these structures is now unquestioned given the wide range of degenerative diseases that stem from *LMNA* gene mutations, including muscular dystrophy disorders, peripheral neuropathies, lipodystrophies, and premature aging syndromes. Here, we review our knowledge about how alterations in nuclear lamins, either by mutation or reduced expression, impact cellular mechanisms that maintain genome integrity. Despite the fact that DNA replication is the major source of DNA damage and genomic instability in dividing cells, how alterations in lamins function impact replication remains minimally explored. We summarize recent studies showing that lamins play a role in DNA replication, and that the DNA damage that accumulates upon lamins dysfunction is elicited in part by deprotection of replication forks. We also discuss the emerging model that DNA damage and replication stress are “sensed” at the cytoplasm by proteins that normally survey this space in search of foreign nucleic acids. In turn, these cytosolic sensors activate innate immune responses, which are materializing as important players in aging and cancer, as well as in the response to cancer immunotherapy.

## Nuclear lamins and compartmentalization of genome function

### Structure of nuclear lamina

**Figure 1.**
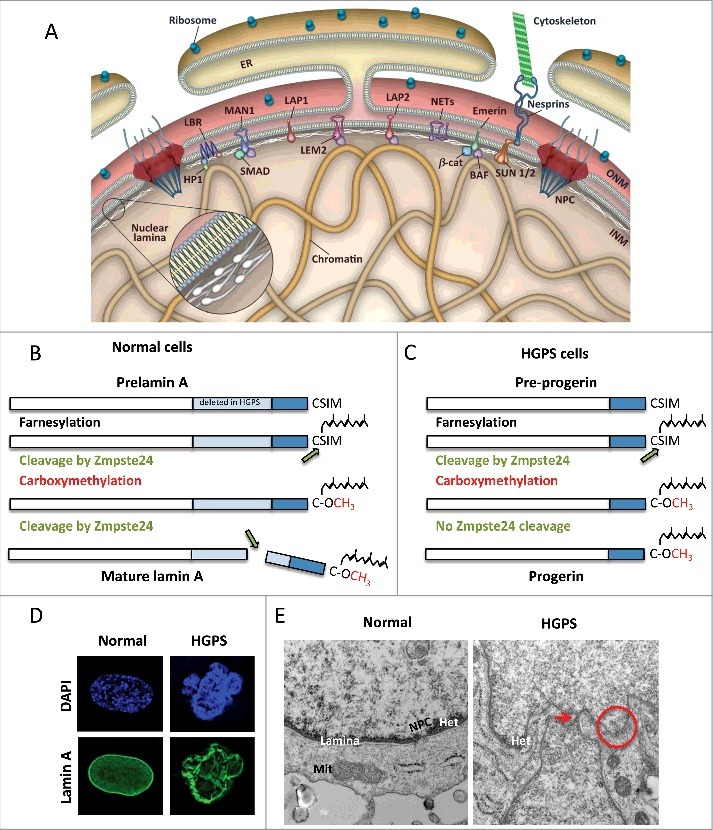
A-type lamins structure and post-translational processing are important for nuclear stability. (A) Schematic representation of the nuclear envelope. The nuclear lamina is a network of intermediate filaments underneath the inner nuclear membrane (INM) that associates with chromatin and INM proteins. Adopted from Dobrzynska et al., *Nucleus* 2016. (B) Lamin-A is synthesized as a pre-lamin-A precursor that undergoes processing of its C-terminus to render mature lamin-A. The -CAAX motif undergoes farnesylation, cleavage of the last three amino acids, followed by carboxyl methylation of the terminal cysteine. This farnesylated form of the protein has high affinity for the inner nuclear membrane.  A second cleavage by the metalloprotease Zmpste24 removes 15 amino acids, including the farnesylated and methylated cysteine, rendering mature lamin-A. (C) HGPS patient-derived cells carry mutations in the *LMNA* gene that cause aberrant splicing of the gene and deletion of 50 residues that include the Zmpste24 cleavage site. This permanently farnesylated and methylated truncated form of lamin-A is known as progerin. (D) HGPS patient-derived fibroblasts cells exhibit profound nuclear morphological abnormalities, as shown by immunofluorescence with lamin-A antibody. (E) Normal and HGPS fibroblasts were processed for electron microscopy to detect structural defects in nuclei.  In normal fibroblasts, note how the nuclear lamina is located underneath the nuclear membrane, and associated with heterochromatin domains (dark staining). A nuclear pore complex is also evident. In HGPS cells, nuclear morphological abnormalities such as protrusions and invaginations (arrow), and sites of nuclear membrane discontinuity (circle) are evident.

The nuclear lamina is an intricate meshwork of A-type and B-type lamins, a special class of intermediate filaments that, together with lamin-associated factors, are located underneath the inner nuclear membrane (Figure 1A). Lamins feature a central α-helical rod domain flanked by non-helical globular head and tail domains. The rod domain mediates formation of filaments, while the tail and head domains are involved in protein-protein and protein-DNA interactions [1]. Cryo-electron tomography studies reported that nuclear lamins assemble into tetrameric filaments that appear as globular-decorated fibers of 3.5 nm thickness [2]. The association of lamins into these “high-order structures” results in a scaffold that maintains nuclear architecture and stability [3–7]. The mammalian genome encodes two different types of nuclear lamins: A-type lamins, including lamin-A and –C, which are splice variants of the *LMNA* gene, and B-type lamins, B1 and B2, encoded by *LMNB1* and *LMNB2* genes, respectively [1,8]. Minor isoforms produced by lamin genes include CΔ10, C2, and B3. While B-type lamins are found in all types of cells, lamin-A/C are expressed in differentiated cells and found both at the nuclear periphery and throughout the nucleoplasm. Lamin-C is directly translated from the *LMNA* mRNA into the mature form. Lamin-A is initially synthesized as a pre-lamin-A form that undergoes extensive post-translational processing to produce the mature form (Figure 1B). Pre-lamin-A carries a C-terminal –CAAX motif that is first farnesylated and subsequently cleaved by the Zmpste24 enzyme. The proteolytic cleavage removes the last three residues of the –CAAX motif, exposing the terminal cysteine to carboxyl methylation. A second cleavage event by Zmpste24 removes the C-terminal 15 amino acids, producing the mature form of lamin-A [9].

Extensive evidence demonstrates that mutations that prevent proper maturation and post-translational processing of pre-lamin-A lead to disease. One of the most severe laminopathies, Hutchinson Gilford Progeria Syndrome (HGPS), is caused by a *de novo* single-base substitution in the exon 11 of *LMNA* gene (Figure 1C). This mutation activates a cryptic splice site that leads to an in-frame deletion of 50 residues near the C-terminus of pre-lamin-A. The deleted sequence includes the recognition site of the Zmpste24 enzyme. This results in a permanently farnesylated and carboxyl methylated pre-lamin-A protein known as “progerin”. The expression of this mutant protein over time causes malfunction of multiple nuclear processes, ultimately leading to cellular and organismal aging [10–12]. As evidence of the toxic effects of progerin, HGPS patient-derived cells exhibit nuclear morphological abnormalities, loss of heterochromatin from the nuclear periphery, genome instability and premature senescence [13–15] (Figure 1D,E).

### Functions of A-type lamins

Lamins provide structural support to the nucleus, with their level of expression directly linked to nuclear stiffness and tissue rigidity and plasticity [16–21]. Nuclear stiffness is important for the viability and correct function of all cell types, especially in tissues subjected to strong mechanical tension such as muscle and skin, as well as for migrating cells [16,20,22]. Other important functions of nuclear lamins include: (1) assisting nuclear envelope dissolution during mitosis by becoming soluble upon phosphorylation [23,24]; (2) transducing signals from the cytoskeleton into the nucleus via direct contact with nuclear pore complexes, and controlling nuclear trafficking [25–27]; (3) regulating gene expression via modulation of epigenetic mechanisms [15,28,29] and interaction with transcription factors at both active and inactive genes [30,31]; and (4) ensuing genome stability via modulation of mechanisms of DNA repair and telomere homeostasis [32–37]. Therefore, lamins are thought of as genome caretakers. However, the precise molecular mechanisms orchestrated by nuclear lamins in most of these processes are still largely unknown and the object of intense research. Perhaps the most studied function of lamins so far has been that of tethering chromatin to specific sub-nuclear regions.

### A-type lamins: Chromatin anchors

Physical mapping approaches determined that the nuclear lamina is directly associated with chromatin [38–40]. Accordingly, global chromatin association profiles revealed that ∼40% of the human genome is organized into ∼1300 lamin-associated domains (LADs) spanning from a few Kb to more than 10Mb [6,39,41]. LADs have heterochromatic features characterized by low gene density, minimal transcriptional activity, and repressive chromatin marks such as H3K27me3 and H3K9me2. These domains are found in several cell types and occupy conserved positions in human and mouse nuclei. In addition, there are hundreds of genomic regions known as “facultative LADs” that change association with lamins during differentiation [6]. The biology of LADs is still poorly understood and is the subject of intense investigation.

The signatures that drive association of specific chromatic regions with lamins are still unknown. However, a number of nuclear factors have been proposed to cooperate with lamins in tethering DNA to the nuclear periphery. Candidates include lamin-associated proteins 2α and 2β (LAP2α and LAP2β), the nuclear membrane protein Emerin, the transcriptional regulators cKrox and YY1, the histone deacetylase HDAC3, and a number of less characterized inner nuclear transmembrane proteins named NETs [42]. Importantly, the factor LAP2α has been shown to mediate the physical interaction of lamins with euchromatic regions [43,44]. These studies suggest that lamins engage in both direct and indirect interactions with chromatin, implying a certain plasticity of the nuclear lamina in the nuclear space. Therefore, the role of lamins at the nuclear periphery goes beyond that of simply serving as chromatin anchors.

In addition, recent studies directly show that nuclear lamins are also involved in determining gene positioning [40]. Specifically, the authors monitored nuclear positioning of LADF, a peripheral non-coding LAD, and of two centrally localized genes *COL1A1* and *OR5H1*, which are transcriptionally active and inactive, respectively. Depletion of lamins shifted LADF from the periphery toward the center of the nucleus. Although smaller, a similar effect was also observed for *COL1A1* and *OR5H1* that underwent repositioning toward the periphery. The authors also identified 50 novel factors involved in gene positioning. Most of them are components of the replication and post-replication chromatin assembly machinery. Accordingly, shifts in gene positioning require progression through the S-phase of the cell cycle but not through mitosis [40]. Interestingly, gene positioning is driven by DNA replication *per se* rather than by the binding of specific positioning factors, as shown in growth-arrested cells where knocking down several of these proteins did not affect gene positioning. This is a remarkable observation as lamins, not by chance, dissolve during mitosis while being still present during S-phase, when they seem to be required for proper DNA replication [33,45]. Overall, these studies suggest that lamins, along with multiple factors and molecular pathways, actively participate in maintaining the proper compartmentalization of genome function.

### A-type lamins maintain proper nuclear dynamics

Previous studies showed that A-type lamins play a critical role in nuclear compartmentalization of telomeres [35]. Typically, mammalian telomeres are distributed throughout the entire nucleoplasm in interphase, while they assemble at the center of the nucleus during mitosis [46]. We found changes in nuclear distribution of telomeres in interphase nuclei upon ablation of lamin-A/C, with telomeres accumulating towards the nuclear periphery [35]. Three-dimensional tracking of telomeres trajectories showed that these genomic loci do not occupy a fixed position, but rather they are subjected to transient anomalous diffusion [47]. Unlike normal diffusion that is fast, anomalous diffusion is a slow and localized motion indicative of interactions among genomic regions and constituents of the nucleoplasm. Early studies from de Lange's group suggested that telomeres could interact with components of the nuclear matrix, although at that time a link with nuclear lamins was not established [48]. Recent *in vivo* imaging studies have brought to light that lamin-A/C is critical to coordinate nuclear dynamics of genome loci [49,50]. Upon depletion of lamin-A/C, there is a remarkable transition of telomeres, as well as centromeres and other chromosomal loci, from slow-anomalous to fast-normal diffusion. This was accompanied by a drastic increase in the area explored by each of the genomic regions monitored [49]. Therefore, in absence of lamin-A/C, genomic loci experience increased mobility, which translate into a higher likelihood of being engaged in promiscuous interactions. The repercussions that unscheduled nuclear dynamics might have on DNA transactions and on genome stability are limitless, and to date, remain poorly understood.

## Lamins-related diseases: Laminopathies and cancer

### Laminopathies

Consistent with the multitude of cellular functions exerted by lamins, mutations in *LMNA* gene and lamin-interacting factors are associated with a broad spectrum of disorders [51–57] (Table 1). Laminopathies are among the most intriguing degenerative disorders across human pathologies, with the relationships between genotypes and phenotypes remaining poorly understood [58,59]. Some hotspot mutations in the *LMNA* gene have been associated with specific types of laminopathies, as in HGPS. However, it is noteworthy that different mutations throughout the *LMNA* gene can cause the same type of disorder, and different substitutions of the same nucleotide can elicit different pathologies. In addition, the same *LMNA* mutations can have different penetrance between individuals, which sometimes translates into lack of symptoms or failure to ascribe them to laminopathies [60]. Interestingly, in each laminopathy only a single or a few tissues are compromised though lamins are ubiquitously expressed.

**Table 1.** Tissue specific phenotypes caused by LMNA gene mutations. Table shows degenerative disorders that are associated with mutations in the *LMNA* gene. Mutations can affect specific tissues such as striated muscle (skeletal and cardiac) in the case of EDMD, or adipose tissue in the case of FPLD. Other laminopathies affect peripheral nerves only, such is the case of Charcot-Marie-Tooth type 2 peripheral neuropathy. There are also laminopathies that affect multiple tissues and systems such is the case of HGPS. Progeria kids present with growth retardation, alopecia, micrognathia, skin moulting, reduced subcutaneous fat that is widespread, and premature vascular occlusive disease, which can cause stroke and death.

Disease	Clinical symptoms
Emery-Dreifuss Muscular Dystrophy EDMD	Muscle contractures in elbows, Achilles tendoms and posterior neck; wasting of skeletal muscle; dilated cardiomyopathy with conduction defects
Limb girdle muscular dystrophy 1B LGMD1B	Slowly progressing wasting of shoulder and pelvic muscles due to necrosis, cardiac disturbances
Dilated cardiomyopathy DCM	Ventricular dilation and systolic dysfunction sometimes accompanied by conduction defects
Familial partial lipodystrophy FPLD	Loss of fat tissue from extremities, accumulation in neck and face, insulin-resistant diabetes, hyperlipidemia, atherosclerotic vascular disease
Generalized lipoatrophy	General lipodystrophy, insultin-resistant diabetes, progeroid features
Charcot-Marie Tooth disorder 2B	Neuromuscular disease; axonal degeneration, sensory impairment
Hutchinson-Gilford Progeria Syndrome HGPS	Features of premature aging, short stature, early thinning of skin, loss of subcutaneous fat, premature atherosclerosis and cardiac failure leading to death
Atypical Werner´s Syndrome AWS	Features of premature aging, affects young adults
Mandibuloacral dysplasia A MADA	Skull and face anomalies, skeletal abnormalities, partial lipodystrophy, premature aging symptoms
Restrictive dermopathy RD	Generalized lipodystrophy, intrauterine growth retardation, tight and rigid skin, neonatal mortality

In an effort to explain this complex puzzle, different models have been proposed [52,53]. According to the “gene expression model,” different *LMNA* gene mutations would affect the position or expression of different genes, leading to tissue-specific laminopathies. The “mechanic model” proposes instead that alterations in nuclear lamina would affect the mechanical properties of the nucleus and impact tissues subjected to strong mechanical tensions such as muscles and skin. Other models suggest that tissue degeneration in laminopathies arises from defects in the ability of progenitor cells to differentiate along specific lineages, and an overall exhaustion of stem cell compartments due to tissue damage over time [61–63]. Based on the tissue(s) affected, laminopathies can be grouped as muscular dystrophies, peripheral neuropathies, lipodystrophies, as well as diseases that affect a variety of tissues such as Atypical Werner Syndrome (AWS), Restrictive Dermopathy (RD), and Hutchinson Gilford Progeria Syndrome (HGPS). Understanding the factors that determine laminopathy severity as well as why mutations affect different tissues is an active area of investigation.

### A-type lamins and cancer

Genome instability and “nuclear atypia” are hallmarks of cancer, with the latter being widely used by pathologists in the assessment of tumor grade [64]. Because of the role of nuclear lamins in maintaining nuclear morphology, changes in the expression levels of these structural proteins have long been monitored in cancer. In fact, changes in lamin-A/C expression are common in human tumors [22,65]. Lamins participate in many pathways with tumor suppressive or oncogenic function, and also play a role in both promotion and inhibition of apoptosis. Perhaps the best connection between lamins and cancer is the evidence that A-type lamins are essential for the stability of the retinoblastoma tumor suppressor proteins pRb and p107 [35,66]. These and other findings have placed lamins on the spotlight as proteins highly relevant for understanding tumorigenesis [22,65].

Recent studies propose that the role of nuclear lamins in human malignancies is most likely context-dependent. For instance, decreased expression of nuclear lamins has been found in prostate, breast, colon, ovarian, and gastric cancers, and is often associated with poor prognosis [65,67,68]. Other studies on colorectal, prostate, and breast cancers reported an association between increased expression of lamin-A/C and better clinical outcomes [64,67]. In lymphoma and leukemia, and in a subset of neuroblastoma cells, hypermethylation of CpG islands at the *LMNA* gene promoter lead to reduced lamin-A/C expression [69]. Thus, lamin-A/C expression is restored by treatment with demethylating agents. Interestingly, reconstitution of lamin-A/C expression reduces cell growth and impairs migration, invasion, and anchorage-independent growth, while progerin expression induces senescence in these neuroblastoma cells. Furthermore, lamin-A/C depletion in unmethylated neuroblastoma cells exacerbates their tumoral properties [69]. This is one clear example whereby alterations in the levels or structure of nuclear lamins impact cellular mechanisms of malignancy. Further studies are needed in different cancer models to determine the importance of epigenetic silencing of the *LMNA* gene in tumorigenesis and the potential of this mechanism as target for anti-tumoral strategies.

In addition, loss of lamins potentiates cancer cell migration through narrow spaces, suggesting a potential role in metastasis [16,20,22,70]. Conversely, in other scenarios, loss of nuclear integrity enhances cancer cell susceptibility to mechanical forces such as the fluid shear stress of the circulatory system, and increases apoptosis [22,70]. Altogether these studies undoubtedly associate lamins with cancers, though the precise impact of lamins on malignancies remains unknown. Moreover, because of the variability among tumor subtypes, it is still controversial whether the expression levels of lamin-A/C can be used as a diagnostic biomarker for cancer.

Given the association between aging and cancer, it is paradoxical that HGPS patients do not develop cancer despite the high levels of DNA damage and accelerated aging. It was first thought that low cancer risk in these patients was due to their shortened lifespan. However, recent evidence indicates that progerin expression might actually protect HGPS cells from malignant transformation [71]. Genome-wide RNAi screening identified the bromodomain protein BRD4 as a mediator of the oncogenic resistance of HGPS cells. This factor modulates gene expression and has been shown to play both cancer-promoting and anti-metastatic roles, depending on the context. Interestingly, in HGPS cells, BRD4 exhibits altered genome-wide binding patterns leading to inhibition of oncogenic differentiation [71]. However, an association between progerin expression and increased tumorigenesis has been reported for a number of human cancer cells [72]. Importantly, progerin is produced during aging in the normal population [73]. Therefore, the significance of progerin expression during aging of normal cells, as well as the role exerted by this toxic protein in cancer remains to be defined.

Overall, these studies show that disruption of the nuclear lamina has severe repercussions for cellular health. Expression of progerin leads to decline of multiple cellular processes, ultimately resulting in cellular senescence and premature aging. On the other hand, alterations in expression of lamins are often observed in a variety of human malignancies. Aging and cancer are two different faces of genome instability, and disruption of nuclear lamina can contribute to both phenotypes, hence lamins have emerged as critical players in the maintenance of genome stability.

## Lamins as caretakers of the genome

### Role of A-type lamins in DNA repair

Repair of damaged DNA is imperative for maintenance of genome stability. Among the various types of DNA damage, double-strand breaks (DSBs) are certainly the most harmful, as their inefficient or inaccurate repair can lead to mutations, loss of genetic material, and chromosomal translocations [74]. DSBs are generated by exogenous sources as well as by free radicals produced during cellular metabolism, or derive from DNA adducts/abnormal DNA structures during DNA replication. Cells promptly respond to DSBs by activating the DNA damage response pathway (DDR), which involves a plethora of factors that sense, signal, and ultimately repair DNA lesions [37,74].

**Figure 2.**
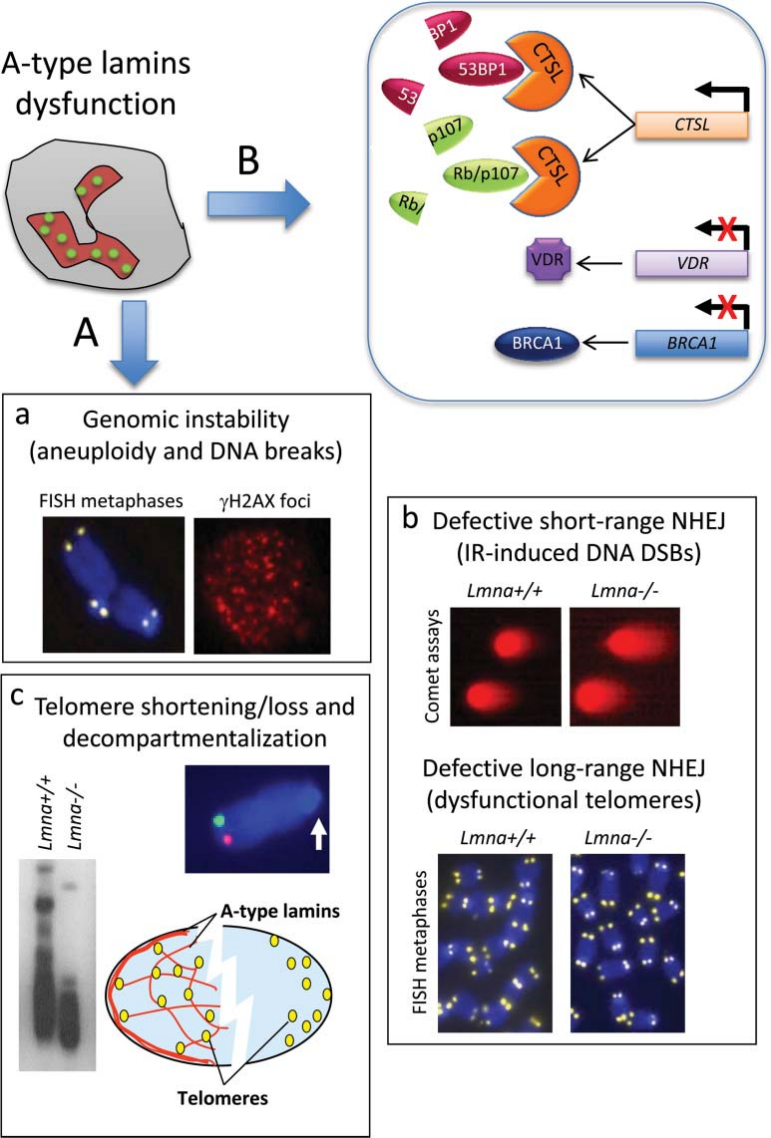
A-type lamins as caretakers of the genome.  (A) Hallmarks of genomic instability that have been reported in A-type lamins-deficient cells include: (a) Aneuploidy and increased frequency of chromosome/chromatid breaks (as shown by telomere-FISH on metaphase spreads), which result in accumulation of DNA damage (γH2AX foci by immunofluorescence); (b) Defects in short-range and long-range NHEJ mechanism of DNA DSB repair, as shown by neutral comet assays performed after ionizing radiation-induced DNA DSBs (short-range), and by FISH on metaphases induced to undergo NHEJ of dysfunctional telomeres (long-range); (c) Telomere shortening (as assessed by Telomere Restriction Fragment analysis), or complete loss, and decompartmentalization of telomeres in the 3D nuclear space (as shown by 3D-telomere-FISH). (B) At a molecular level, reduced expression of A-type lamins causes transcriptional upregulation of *CTSL* gene, and increased global cathepsin L (CTSL) activity. CTSL is responsible for the degradation of 53BP1, and the Rb family members pRb and p107, which are key factors in DNA repair and cell cycle regulation, respectively. The decrease in 53BP1 underlies defects in short-range and long-range NHEJ. In addition, lamins deficiency elicits downregulation of vitamin D receptor (*VDR*) and *BRCA1* gene expression, events that impair HDR repair. Other factors are likely to contribute to genomic instability in different laminopathies such as accumulation of XPA at DSBs, deficiency in DNA-PK holoenzyme function, increased generation of ROS, and epigenetic alterations (not shown in the model).

Different studies have demonstrated that A-type lamins play a role in DNA DSB repair by both non-homologous end joining (NHEJ) and homology directed repair (HDR) [14,34–37]. Under basal conditions, *Lmna*-KO mouse embryonic fibroblasts (MEFs) feature increased aneuploidy, high frequency of chromosome and chromatid breaks, accumulation of high levels of DNA damage (γH2AX), as well as defects in telomere structure, length, and function (Figure 2A). In addition, lamin-deficient cells are hindered in long-range NHEJ of dysfunctional telomeres and in repair of short-range DSBs induced by ionizing radiation [34–36]. These deficiencies are not due to impaired activation of the DDR, but rather to changes in the expression and stability of key factors in DNA repair, such as loss of 53BP1 protein (p53-binding protein 1). Accordingly, reconstitution of 53BP1 rescues both long-range and short-range NHEJ [34].

The observation that 53BP1 protein levels are reduced in lamins-depleted cells suggested the involvement of proteases in the regulation of this important DNA repair factor. We found that lamin-A/C-deficient cells upregulate expression of the cysteine protease cathepsin-L (CTSL) [34], which is responsible for the degradation of 53BP1 (Figure 2B). Increased CTSL activity also leads to degradation of retinoblastoma family members pRb and p107. The loss of these important tumor suppressor proteins could predispose lamins-deficient cells for malignant transformation. Importantly, inhibition of CTSL activity directly via CTSL inhibitors and shRNA-mediated CTSL depletion, or indirectly via vitamin D treatment, results in stabilization of 53BP1 and amelioration of NHEJ defects in lamin-deficient cells. In addition, depletion of lamin-A/C results in transcriptional downregulation of BRCA1 (breast cancer susceptibility gene 1), and impaired HDR, in some cell types [36,75] (Figure 2B). BRCA1 deficiency in laminopathies has been linked to reduced expression of vitamin D receptor (VDR). There is evidence that in lamins-depleted cells, reduced VDR levels contribute to downregulate BRCA1 expression, which in turn causes DNA repair defects. Upon vitamin D treatment, increased expression of VDR and BRCA1 reduces the extent of DNA damage and genomic instability induced by lamins dysfunction [34]. This new link between VDR signaling and DNA repair should be further explored, as vitamin D/VDR deficiency has been implicated in the pathophysiology of aging and aging-related diseases.

In addition to lamins loss, expression of mutant lamins elicits genomic instability. For instance, progerin-expressing cells exhibit delayed recruitment of DNA repair factors 53BP1 and RAD51 to γH2AX-labeled DNA repair foci [76], and an aberrant accumulation of Xeroderma Pigmentosum group A (XPA), a key factor in nucleotide excision repair (NER). XPA binding to DNA lesions activates ATM- and ATR-dependent signaling that contribute to proliferation arrest [76,77]. Interestingly, siRNA-mediated depletion of XPA in progeria cells partially restores recruitment of DNA repair factors to DNA damage sites. Other mechanisms that impair recruitment of DNA repair factors and DNA damage accumulation in laminopathies include reduced levels of the DNA-PK holoenzyme [78], a key factor in NHEJ repair, and defects in chromatin-modifying activities such as the NuRD complex and the histone acetyltransferase Mof, which facilitate DNA repair [79,80].

### Role of A-type lamins in telomere maintenance

The association of lamin-A/C with telomeres, putatively via telomere-binding proteins [81,82], is important for the localization and mobility of telomeres within the 3D nuclear space, as well as for the maintenance of telomere length homeostasis [35,47,50,83]. *Lmna^−^^/^^−^* mice exhibit decreased telomere length when compared to *Lmna^+/+^* mice, as well as an increased frequency of chromosomes lacking telomere signals [35] (Figure 2A). As additional evidence for a role of lamins in telomere biology, proliferative defects on human fibroblasts expressing lamin-A mutants are rescued by telomerase [84]. HGPS fibroblasts are also characterized by faster telomere attrition during proliferation [85,86]. In addition, A-type lamins participate indirectly in the aberrant processing of dysfunctional telomeres by NHEJ, which leads to chromosome end-to-end fusions. Overall, these studies provide a strong correlation between lamins dysfunction and a hindered ability to maintain mechanisms that ensure genome stability.

### DNA repair factors as guardians of replication forks

**Figure 3.**
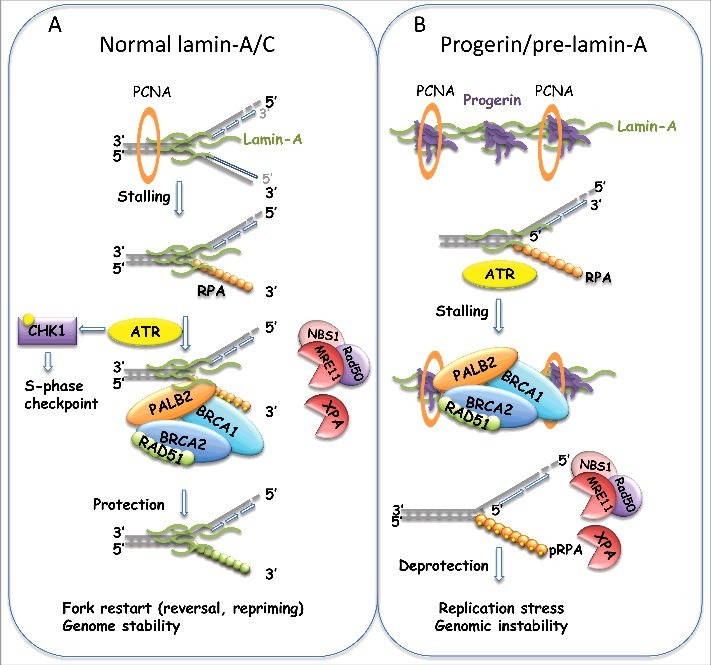
Recruitment of proteins to the replication fork. (A) Scheme of events taking place at stalled forks to protect DNA from nucleolytic degradation and to ensure proper restart of the fork (see text). Proteins with function in DNA DSB repair play a protective role at the replication fork. We propose a model whereby A-type lamins participate in replication fork stability by facilitating the recruitment of proteins that protect the fork from degradation. (B) Studies suggest that progerin or pre-lamin-A sequester PCNA away from the replication fork. It is possible that progerin also hinders the association of protective factors to the fork, leading to fork deprotection, nuclease-mediated degradation, and replication stress. The combination of new technologies such as DNA fibers, iPOND, and electron microscopy will be necessary to test these models.

Emerging evidence suggests that a number of DNA repair factors including 53BP1, RAD51, and BRCA1/2 proteins play DNA repair-independent roles during DNA replication [87]. DNA lesions caused by endogenous and exogenous agents, secondary DNA structures, and transcribing RNA polymerases, pose a challenge for the replication fork [88]. In response to these challenges, replication forks stall and recruit factors that protect them from excessive nucleolytic degradation, while facilitating the restart of the fork to preserve genome stability (Figure 3A). Replication stalling causes uncoupling of the replicative DNA helicase from the DNA polymerase machinery with consequent formation of ssDNA that is promptly coated by RPA (replication protein A complex) [89,90]. The resulting RPA-ssDNA filaments activate the kinase ATR (ATM and Rad3 related) that in turn phosphorylates RPA at multiple sites. At the same time, ATR activates a downstream signaling cascade that culminates with the recruitment to stalled forks of protecting factors such as BRCA1/2 [90], as well as Fanconi Anemia (FA) proteins, which are essential mediators of inter-strand crosslink repair [91]. Protection of the newly synthesized DNA is extremely important, as it prevents stalled forks from being preyed upon by nucleases and translated into DSBs. The most studied nuclease involved in degradation of unprotected stalled forks is MRE11 [92–94]. BRCA1/2 proteins in concert with FA factors chaperone the switch of RPA with RAD51 at ssDNA, blocking MRE11 from inducing genome instability [93]. The loading of RAD51 mediates fork reversal, an important mechanism that enables the fork to pause and then resume without chromosomal breakage [95–99]. Mechanisms regulating reversal and restart of replication forks are only beginning to be unveiled.

Therefore, it emerges from these studies that DNA repair proteins associate with the replication fork during S-phase to avoid DNA damage that might arise from roadblocks encountered by the replisome (Figure 3A). In addition, 53BP1 protein has been shown to protect under-replicated genomic loci, primarily common fragile sites, in cells that continue their progression through G2/M phases of the cell cycle [100]. After entry into mitosis, a fraction of these under-replicated loci generates DNA lesions that are transmitted to daughter cells. Accumulation of 53BP1 at these lesions (53BP1 nuclear bodies) during G1 phase has been proposed to shield chromosomal loci prone to replication stress to facilitate the repair process [101].

### Nuclear lamins and DNA replication

Although lamins have been strongly linked to the maintenance of genome stability, few studies have attempted to characterize the effects of disruption of lamins function on DNA replication. Early studies of replication with *Xenopus* extracts showed that disruption of the nuclear lamina alters the distribution of Proliferating Cell Nuclear Antigen (PCNA) and the Replication Factor Complex (RFC), important factors in the elongation phase of DNA replication, which appear within intranucleoplasmic lamin aggregates [102]. These defects were accompanied by a marked reduction in DNA replication. A subsequent study showed that altered lamin organization inhibits chain elongation in a dose-dependent manner [45]. In addition, co-localization of A- and B-type lamins with PCNA associate these structural proteins with early and late sites of DNA replication, respectively [4,33,103–105] (Figure 3A). These studies suggest a role for lamins in the spatial/temporal organization of replication, which would be in line with the concept that lamins keep the nucleus organized. However, direct evidence for loss of lamins causing mis-localization of replication forks in the 3D-nuclear space is lacking.

More recently, it has been reported that lamin-A/C are required for proper re-start of stalled replication forks [106], although this phenotype was not characterized at a mechanistic level. Expression of pre-lamin-A has also been associated with mono-ubiquitination of PCNA and induction of Pol η, two hallmarks of replication fork stalling [107]. This study presents indirect evidence suggesting that pre-lamin-A mitigates the interaction of PCNA with mature lamin-A, leading to replication fork stalling. In addition, Tang el al. reported that RFC1 is aberrantly degraded in HGPS cells by a serine protease. This cleavage results in defective loading of PCNA and Pol δ onto DNA for replication [108]. Along the same lines, Wheaton et al. showed that progerin binds to PCNA, altering its distribution away from replicating DNA [109]. Moreover, an unbiased screen to identify lamin-A- and progerin-interacting proteins by mass-spectrometry revealed increased interaction of PCNA with progerin compared to lamin-A [110]. These findings support the notion that mutant lamins, and especially pre-lamin-A and progerin, might sequester sufficient PCNA away from the replication fork to elicit replication stress (Figure 3B). Consistent with this idea, XPA accumulates at stalled or collapsed replication forks in progerin-expressing cells, concurrent with a significant loss of PCNA at the forks. Interestingly, depletion of XPA or progerin each restored PCNA at replication forks, while reducing the extent of apoptosis induced by progerin [111]. Thus, progerin expression seems to alter the binding to replication forks, not only of PCNA, but also of factors such as XPA that participate in the repair of DNA lesions. Altogether, these studies suggest that nuclear lamins play a role in DNA replication, and that abnormal proliferation and genomic instability in cells with disrupted lamins function could be the result, at least in part, of increased replication stress. Despite these findings, our understanding of the specific mechanisms whereby lamins ensure proper DNA replication is limited.

The function of lamins in DNA replication could be direct, by binding to the replisome and facilitating replication fork progression and stability, or indirect, by regulating the levels of expression of factors with key roles in replication. Mutant lamins, and especially progerin, could act as an obstacle to the progression of the replication fork by inducing mis-localization of factors that are known to associate with the replisome. In addition, lamins dysfunction could hinder the proper recruitment of replication fork protective factors upon fork stalling, causing replication stress-induced genomic instability (Figure 3B). To understand in-depth how lamins dysfunction affects DNA replication requires the utilization of newly developed techniques such as single-molecule replication assays (DNA fiber assays), iPOND (Isolation of Proteins On Nascent DNA) [112], and electron microscopy [113], as well as depletion/reconstitution experiments to find strategies that rescue replication defects.

Our recent studies performing DNA fiber assays have begun to decipher specific mechanisms whereby disruption of nuclear lamins impact DNA replication. In particular, we find that progerin expression, but not overexpression of lamin-A, causes replication stress, characterized by increased replication fork stalling in the absence of replication inhibitors [114]. In addition, stalled replication forks are deprotected and subjected to nuclease-mediated fork degradation. As such, inhibition of MRE11 nuclease rescues replication defects in progerin-expressing cells. Moreover, in line with the beneficial effect of vitamin D rescuing genomic instability in laminopathies, we discovered that vitamin D treatment ameliorates replication fork deprotection and replication stress in progerin-expressing cells [114]. Although the molecular mechanisms underlying vitamin D's effect rescuing replication stress in progerin-expressing cells remain to be characterized, this finding has important implications from a therapeutic perspective and for delineating the importance of replication stress to the progeria phenotype.

## Lamins dysfunction activates innate immune responses

### Genomic instability activates innate immune responses

A model is emerging whereby DNA damage and replication stress elicit the activation of inflammatory responses that contribute to tumorigenesis in some contexts, and senescence/aging in others [115,116]. Inflammation is triggered when innate immune cells detect infection or tissue damage. A delicate balance in the activation of inflammatory responses, especially the NFκB (nuclear factor kappa B) and IFN (interferon) pathways, is essential for health: insufficient activation results in susceptibility to pathogen infection, while excessive activation leads to autoimmune diseases. The first barrier to infection is the recognition of microbial features by the host cell [117]. Pattern-recognition receptors (PRRs), proteins that survey extracellular and cytoplasmic spaces for the presence of pathogens, activate immune responses upon detecting foreign nucleic acids [117–119]. These include cytosolic sensors of RNA (RIG-1, MDA5, OAS, and PKR) and DNA (DAI, IFI16 and cGAS), and the Toll-like receptor (TLR) family that resides in endolysosomes. PRRs signal through mediators (MAVS, STING, MyD88, TRIMs, and TRAF complexes) to activate NFκB and IFN-regulatory factors IRF3 and IRF7, which translocate to the nucleus and activate transcription of IFNs and cytokines. Secreted IFNs trigger a signaling cascade that activates STAT1 (signal transducer and activator of transcription 1) and the ISGF3 complex, composed of STAT1, STAT2 and IRF9, which induces the expression of hundreds of IFN-stimulated genes (ISGs) involved in the antimicrobial response [120]. Importantly, STAT1 and downstream ISGs can also be activated directly via PRRs in an IFN-independent fashion [121,122].

The activation of innate immune responses also occur in the absence of infection, when cells erroneously recognize self nucleic acids as foreign [123]. Self-DNA is sequestered in mitochondria and nuclei, away from cytoplasmic sensors of nucleic acids. However, in certain conditions, these sensors come in contact with self-DNA. For example, DNA from neighboring damaged cells is engulfed by endolysosomes, where TLRs reside [124]. PRRs can also recognize tumor-derived antigens, and recent evidence indicates that they play an active role in the recognition of malignant cells by the immune system. Accordingly, PRRs such as TLRs, RIG-I, cGAS, and STING are emerging as promising new targets to improve cancer immunotherapy [125]. Elegant studies also showed that DNA breaks are “endogenous danger signals” that trigger a basal IFN response. Fibroblasts from Ataxia Telangiectasia (AT) and Fanconi Anemia (FA) patients, lacking key DNA repair factors, accumulate DNA fragments in the cytoplasm that are recognized by PRRs, inducing an inflammatory response [126,127], which can promote senescence and inhibit stem cell function [116]. Similarly, telomere and mitochondrial dysfunction, and genotoxic insults can trigger activation of PRRs, priming the innate immune response [116,128,129]. Interestingly, IFN pathway inactivation extends lifespan in telomerase knockout mice, linking IFN signaling and premature aging [116]. In addition, replication stress caused by inhibitors of replication or by depletion of replication fork protective factors, cause accumulation of self-nucleic acids in the cytoplasm. These self-nucleic acids are recognized as foreign by PRRs, leading to activation of immune responses [130–132]. For instance, the cGAS/STING cytosolic DNA sensing pathway activates IFN responses [119], and has been implicated in malignant transformation [133,134], and senescence and aging [135]. In addition, the cGAS/STING pathway is important for intrinsic antitumor immunity [133,136]. In contrast to DNA, RNAs are in abundance in the cytoplasm. Complex editing and processing of host RNAs differentiates them from pathogens' RNAs, hiding them from PRRs and preventing the activation of an IFN response [137–139]. The current view is that cells generate immunogenic nucleic acid species via the accumulation of endogenous DNA/RNA byproducts in the cytosol, as those generated during DNA replication, repair of DNA breaks, telomere dysfunction, or defective RNA editing.

### Activation of innate immune responses upon lamins dysfunction

Previous studies demonstrated that cellular and mouse models of HGPS exhibit inflammation markers, primarily an elevated NFκB transcriptional profile, which is linked to activation of the DDR [62,140–142]. Genetic or pharmacological inhibition of the NFκB pathway slows aging and increases longevity of progeria mice [142], and improves the efficiency of reprogramming of somatic HGPS cells and cells from aged individuals into iPSCs [143], a paradigm of cellular rejuvenation. These data directly implicate NFκB-mediated inflammation in HGPS progression, and suggest that controlling inflammation could help rejuvenate tissues and delay organismal aging in HGPS patients. To achieve this goal requires a deeper understanding of the HGPS inflammatory phenotype, including defining the etiology of inflammation, the factors upstream and downstream of NFκB, as well as additional contributing pathways.

We recently found that replication stress in HGPS patient-derived fibroblasts is accompanied by accumulation of chromatin at the cytoplasm, upregulation of cytosolic sensors of nucleic acids -cGAS, STING, RIG-I, MDA5, and OASs-, and robust activation of a cell intrinsic interferon (IFN)-like response [114]. We present evidence for this IFN-like response, which is regulated by STAT1, contributing to cellular aging phenotypes in progeria cells, such as reduced proliferation and migration capabilities. This finding is notable because STAT1 is a prominent regulator of inflammation in immune and vascular cells during atherosclerosis, and an important therapeutic target for cardiovascular disease [144], the main cause of death of HGPS patients. We envision that progerin expression in vascular cells from HGPS patients could recapitulate the STAT1 pathway activation observed in fibroblasts, being a contributor to the decline of vascular cells characteristic of this disease.

**Figure 4.**
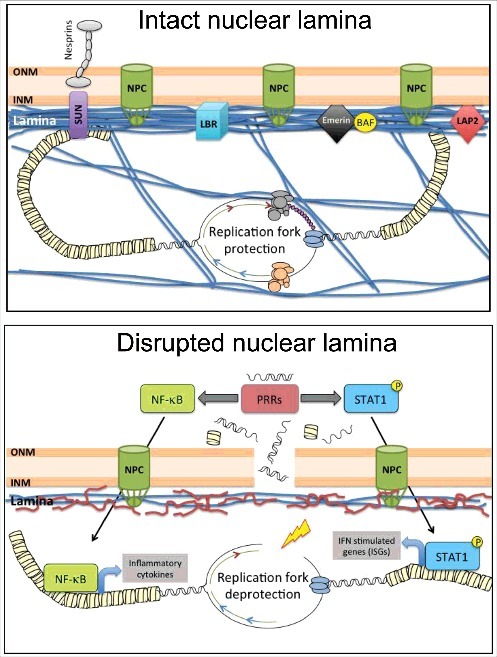
Model of progerin-induced activation of a cell intrinsic IFN-like response. Nuclear lamins and lamin-associated proteins help maintain nuclear architecture and integrity, and genome stability. Hallmarks of progerin-expressing cells include nuclear morphological abnormalities, DNA repair defects, and telomere dysfunction, leading to DNA damage accumulation and premature senescence. In addition, progerin expression hinders replication fork progression, causing replication stress. We propose that replication stress and DNA damage accumulation generates immunogenic nucleic acids that leak outside the nucleus. This “false alarm” of pathogen invasion activates PRRs, including cGAS and STING, and downstream NFkB and STAT1 pathways, which together drive an IFN-like response. Treatment with vitamin D, as well as other compounds such as farnesyltransferase inhibitors and rapamycin, all-trans retinoic acid, and remodelin, rescue replication defects and reduce DNA damage. These improvements are accompanied by repression of the STAT1/IFN-like response, suggesting that this pathway contributes to cellular decline in progeria.

Importantly, we showed that vitamin D treatment, as well as other compounds previously shown to ameliorate disease phenotype like farnesyltransferase inhibitors and rapamycin [145–147], all-trans retinoic acid [21,146,148], and remodelin [149], markedly repress the STAT1/IFN-like response [114]. We propose that in progerin-expressing cells, DNA damage and replication stress, together with disruption of nuclear integrity, leads to leakage of immunogenic nucleic acids into the cytoplasm. This “false alarm” of pathogen invasion would activate PRRs, which in turn activate NFκB and STAT1 pathways, driving an IFN-like response (Figure 4). We also posit that vitamin D, by reducing replication stress and DNA damage, ameliorates the generation of immunogenic nucleic acids that activate the IFN response. Vitamin D could also have a more direct effect in the STAT1/IFN-like response, as previous studies have shown that the vitamin D/VDR axis can regulate STAT1 activation [150]. Defining the causes of this cell-intrinsic IFN response and its consequences for organismal decline in HGPS, as well as the mechanisms underlying vitamin D actions might reveal ways to reduce its pathological impact in HGPS and in normal aging, as progerin is expressed in cells from old individuals [73]. In fact, inflammation is considered a key factor in the pathophysiology of normal aging. The systemic low-grade chronic inflammation that develops as a consequence of aging “inflammaging” poses a high risk for morbidity and mortality in the elderly, and most age-related diseases have an inflammatory component [151–153]. Yet, the etiology of inflammaging remains obscure. Interest in identifying the pathways that cause and control inflammaging has grown recently due to their high potential as targets for improving health in the aging population.

## Concluding remarks

For as much progress as we have made in understanding the role of lamins as genome caretakers, we are really just beginning to uncover the molecular mechanisms underlying genomic instability in laminopathies. Recent findings suggest that, one day, the nuclear lamina might be targeted for treating cancer or slowing normal aging. More imminently, this relationship might be exploited to delay the detrimental pathology of laminopathies. Many questions still remain to be answered with respect to direct and indirect roles of lamins in mechanisms that safeguard genome stability. In addition, how lamins dysfunction-induced changes in genome compartmentalization and mobility in the 3D nuclear space impact DNA transactions such as transcription, replication and repair remains poorly understood. The discovery that replication stress contributes to DNA damage in HGPS is the latest evidence of the importance of an intact nuclear lamina for proper maintenance of genome stability. Further, the finding that replication stress can lead to activation of pro-inflammatory cascades such as NFκB and IFN signaling is another indication that genomic instability is a key contributor to the phenotype of these cells. Specific strategies to target replication stress are now needed both *in vitro* and *in vivo* to determine how replication stress independently contributes to disease phenotypes. In addition, whether replication stress is a common phenotype in lamins-related diseases remains to be tested. Similarly, the mechanisms whereby therapies currently used to ameliorate disease in laminopathies -farnesyltransferase inhibitors, rapamycin, ATRA, remodelin, and vitamin D, among others- reduce replication stress are not known. These certainly are important questions to be addressed in the coming years. Much to learn about lamins function, we still have.

## Abbreviations

*53BP1*: p53-binding protein 1
*AT*: Ataxia Telangiectasia
*ATM*: Ataxia telangiectasia mutated
*ATR*: ATR (ATM and Rad3 related)
*AWS*: Atypical Werner Syndrome
*BRCA1, BRCA2*: breast cancer susceptibility genes 1 and 2
*BRD4*: bromodomain containing protein 4
*cGAS*: cyclic GMP-AMP synthase
*cKrox*: zinc finger transcription factor encoded by *Zpf67* gene
*COL1A1*: encodes the major component of type I collagen
*CTSL*: cathepsin L
*DAI*: DNA-dependent activator of interferon regulatory factors
*DDR*: DNA damage response
*DNA-PK*: DNA dependent protein kinase
*DSBs*: double-strand breaks
*FA*: Fanconi Anemia
*HDAC3*: histone deacetylase 3
*HDR*: homology-directed repair
*HGPS*: Hutchinson Gilford Progeria Syndrome
*IFI16*: gamma-interferon-inducible protein 16
*IFN*: interferon
*iPOND*: isolation of proteins on aascent DNA
*IRF3, IRF7*: interferon regulatory transcription factors 3 and 7
*ISGs*: IFN-stimulated genes
*LAD*: Lamin-associated domain
*LAP2α, LAP2β*: lamin-associated proteins 2α and 2β
*LMNA*: Lamin A gene
*LMNB1, LMNB2*: Lamin B1 and B2 genes
*MAVS*: mitochondrial antiviral-signaling protein
*MDA5*: melanoma differentiation-associated protein 5
*MEFs*: mouse embryonic fibroblasts
*Mof*: histone acetyl transferase
*MRE11*: enzyme with endonuclease and exonuclease activity
*MyD88*: myeloid differentiation primary response gene 88
*NER*: nucleotide excision repair
*NETs*: inner nuclear transmembrane proteins
*NFκB*: nuclear factor kappa B
*NHEJ*: non-homologous end joining
*NuRD*: nucleosome remodeling deacetylase
*OASs*: 2’-5’-oligoadenylate synthetases
*OR5H1*: olfactory receptor family 5 subfamily H member 1
*p107*: retinoblastoma family member
*PCNA*: proliferating cell nuclear antigen
*PKR*: protein kinase RNA activated
*pRb*: retinoblastoma family member, also p105
*PRRs*: pattern-recognition receptors
*RAD51*: recombinase
*RD*: Restrictive Dermopathy
*RFC*: replication factor complex
*RIG-I*: retinoic acid-inducible gene I
*RNAi*: RNA interference
*RPA*: replication protein A complex
*STAT1*: signal transducer and activator of transcription 1
*STING*: stimulator of interferon genes
*STING*: stimulator of interferon gens
*TLR*: Toll-like receptor
*TRAF*: TNF receptor associated factor protein family
*TRIMs*: tripartite motif-containing proteins
*VDR*: vitamin D receptor
*XPA*: Xeroderma Pigmentosum group A
*YY1*: gene that encodes the ying yang 1 transcriptional repressor protein
*γH2AX*: phosphorylated histone variant H2AX
